# Comparative Pathogenesis of an Avian H5N2 and a Swine H1N1 Influenza Virus in Pigs

**DOI:** 10.1371/journal.pone.0006662

**Published:** 2009-08-17

**Authors:** Annebel De Vleeschauwer, Kalina Atanasova, Steven Van Borm, Thierry van den Berg, Thomas Bruun Rasmussen, Åse Uttenthal, Kristien Van Reeth

**Affiliations:** 1 Laboratory of Virology, Faculty of Veterinary Medicine Ghent University, Merelbeke, Belgium; 2 Avian Virology & Immunology, Veterinary & Agrochemical Research Centre, Brussels, Belgium; 3 National Veterinary Institute, Technical University of Denmark, Kalvehave, Denmark; Institute of Molecular and Cell Biology, Singapore

## Abstract

Pigs are considered intermediate hosts for the transmission of avian influenza viruses (AIVs) to humans but the basic organ pathogenesis of AIVs in pigs has been barely studied. We have used 42 four-week-old influenza naive pigs and two different inoculation routes (intranasal and intratracheal) to compare the pathogenesis of a low pathogenic (LP) H5N2 AIV with that of an H1N1 swine influenza virus. The respiratory tract and selected extra-respiratory tissues were examined for virus replication by titration, immunofluorescence and RT-PCR throughout the course of infection. Both viruses caused a productive infection of the entire respiratory tract and epithelial cells in the lungs were the major target. Compared to the swine virus, the AIV produced lower virus titers and fewer antigen positive cells at all levels of the respiratory tract. The respiratory part of the nasal mucosa in particular showed only rare AIV positive cells and this was associated with reduced nasal shedding of the avian compared to the swine virus. The titers and distribution of the AIV varied extremely between individual pigs and were strongly affected by the route of inoculation. Gross lung lesions and clinical signs were milder with the avian than with the swine virus, corresponding with lower viral loads in the lungs. The brainstem was the single extra-respiratory tissue found positive for virus and viral RNA with both viruses. Our data do not reject the theory of the pig as an intermediate host for AIVs, but they suggest that AIVs need to undergo genetic changes to establish full replication potential in pigs. From a biomedical perspective, experimental LP H5 AIV infection of pigs may be useful to examine heterologous protection provided by H5 vaccines or other immunization strategies, as well as for further studies on the molecular pathogenesis and neurotropism of AIVs in mammals.

## Introduction

Pigs are naturally susceptible to influenza A viruses of H1N1, H3N2 and H1N2 subtypes and these viruses are enzootic in swine producing regions worldwide. Most swine influenza viruses are reassortants containing genes from avian, human and swine origin, but the origin and nature of swine influenza viruses differ between continents [1]. This shows that pigs are also susceptible to influenza viruses of human and avian origin. On several occasions low pathogenic (LP) avian influenza viruses (AIVs) belonging to various hemagglutinin and neuraminidase subtypes (H1N1, H3N2, H3N3, H4N6, H5N2, H9N2) have been isolated from pigs in the field [2]–[8]. Highly pathogenic (HP) AIVs, or serologic evidence of infection with these viruses, have also been found in pigs in nature, e.g. during the H7N7 outbreak in The Netherlands in 2003 [9] and during the current H5N1 outbreaks in Asia [10]–[13]. In addition, the susceptibility of pigs to AIVs has been confirmed experimentally. Experimental intranasal inoculation of pigs with most AIVs examined, both LP and HP, generally resulted in moderate virus titers in nasal swabs and seroconversion [9], [10], [14]–[18].

For years it has been thought that pigs are more susceptible to AIVs than humans and that they can serve as intermediate hosts and mixing vessels for the adaptation and/or transmission of AI viruses from birds to humans [18]. Several more recent findings, however, have started to question this hypothesis. Firstly, virtually no AIVs have been able to maintain themselves in the swine population. One exception here is the predominant H1N1 swine influenza virus lineage in Europe, which is of entirely avian origin [1]. Secondly, the incidence of H5N1 virus infection in pigs in Asia appears to be very low when compared to the high numbers of infected birds. In Vietnam, where HP H5N1 is endemic among poultry, only eight out of 3175 sera collected in slaughterhouses in 2004 tested H5N1 antibody positive [10]. Also, no serological evidence of infection with H5 or H9 AIVs was found in 742 serum samples collected from fattening pigs in Korea in 2005–2006 [19]. Thirdly, cases of HP H5 and H7 AIV infection in humans are mostly due to close contact with infected poultry [1] and these viruses also infect other species such as tigers, leopards, stonemartens, cats and dogs [20]–[23]. Fourthly, not all AIVs are able to infect pigs under experimental conditions. In a study of the replication potential of 38 different AIVs in pigs, one fourth of the viruses examined were not excreted, nor did they induce a serological response [15]. Similarly, two H5N1 HPAIVs isolated from chickens in Japan failed to induce a productive infection or seroconversion in pigs [24] and HP H5 and H7 AIVs failed to transmit between pigs [9], [10], [18].

The pathogenesis of swine influenza is well known and resembles that of human influenza [1]. Swine influenza viruses cause an acute infection of the respiratory tract with typical cases exhibiting fever, depression, labored breathing and coughing. Virus replication is mainly restricted to epithelial cells in the respiratory tract with the lung being the major target organ, but the nasal mucosa, tonsils, trachea and tracheobronchial lymph nodes are also involved [25]. Virus excretion in nasal swabs and virus replication in the lungs are short-lasting and limited to the first 6 or 7 days after infection. Experimentally, typical disease can only be induced by intratracheal inoculation of a high virus dose (≥7.0 log_10_ ID_50_) and is unlikely after intranasal inoculation. This is most probably a reflection of the abrupt and massive virus replication in the lungs following intratracheal inoculation, which in turn induces an overwhelming and simultaneous production of several cytokines. Unlike for swine influenza, data about the pathogenesis of AIVs in pigs are scarce. In a study of Choi et al. [10], one pig each time was inoculated intranasally with each of four different H5N1 HPAIVs. Virus replication was mainly restricted to the respiratory tract, i.e. tonsils, trachea and lung. Despite the absence of viremia, two viruses were also recovered from the liver. In all cases, HP H5N1 virus infection passed subclinically. More recently, Lipatov and co-workers [16] concluded that domestic pigs have a low susceptibility to HP H5N1 AIVs after intranasal inoculation of pigs with virus isolates from humans and birds in Asia in 2003–2005. All four viruses examined replicated mainly in the lungs without evidence of systemic infection. Only two H5N1 isolates produced similar lung virus titers as those obtained by H1N1 and H3N2 swine influenza viruses. Only those AIVs were recovered from the upper respiratory tract, though at lower titers than the swine viruses. Clinical signs and respiratory lesions were milder for the AIVs than for the swine viruses and nasal virus excretion was 2 to 3 log_10_ EID_50_ lower and of shorter duration. In both of the above mentioned studies pigs were slaughtered exclusively at 5 or 6 days post inoculation (dpi). Because of the highly acute nature of influenza virus infections, the early time points of infection and the kinetics of an AIV infection in pigs merit investigation.

In this study we aimed to examine the pathogenesis of a LP H5N2 AIV in pigs, covering the complete course of infection. We had two specific aims: 1) to compare the tissue tropism of a LP H5N2 AIV with that of an H1N1 swine influenza virus and 2) to compare the degree of replication of these viruses within the porcine respiratory tract. In addition we wanted to determine whether the avian virus replicates preferentially in the lower respiratory tract of pigs, as has been described for HP H5N1 viruses in pigs [16] and humans [26]. All work was done with a LPAIV since this allowed us to work under biosafety level-2 conditions and to examine large numbers of pigs. Two different inoculation routes were used: the intranasal route to simulate a more natural way of infection and the intratracheal route to pursue a more reproducible inoculation method.

## Results

### Influenza viruses used for inoculation

The Ck/B/99 virus used in this study is representative for LP H5 AIVs circulating in Europe in the late nineties. It has the typical conserved avian amino acid signature in the receptor binding site of the HA (138A, 190E, 194L, 225G, 226Q, 228G; H3 numbering) that was also found in some recent HP H5N1 viruses [16]. The Sw/B/99 virus belongs to the avian-like H1N1 virus lineage which is enzootic in swine populations of Western Europe. This virus is of entirely avian origin but carries amino acid substitutions (T155V, T159N, E190D; H3 numbering) that are considered essential for adaptation of the avian HA to swine [27].

### Intranasal inoculation of pigs with an avian H5N2 or swine H1N1 influenza virus

To compare the virulence of Ck/B/99 and Sw/B/98 after intranasal inoculation all pigs were daily monitored for respiratory and general symptoms. All pigs remained clinically healthy after inoculation with Ck/B/99, whereas two out of six pigs inoculated with Sw/B/99 showed mild respiratory symptoms (sneezing, coughing) at 2 and 3 dpi. At the time of necropsy, gross lesions were only found in the lungs. Well-marked dark-red areas of lung tissue consolidation were seen after inoculation with Ck/B/99 in one out of the two pigs euthanized at each time point. The lesions involved only 3 to 15% of the total lung surface and were mainly seen in the cardiac lung lobe of the right lung. Inoculation with Sw/B/98 induced similar lung lesions (6 to 23% lung involvement) in five out of six pigs, but they were evenly distributed among all lung lobes.

**Figure 1:**
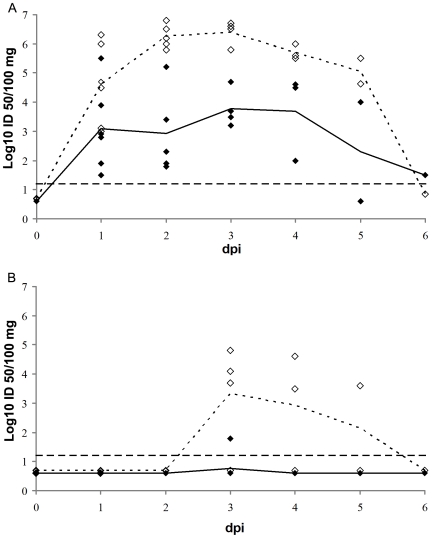
Kinetics of nasal excretion of Ck/B/99 and Sw/B/98 viruses in pigs. Individual virus titers after intranasal (A) or intratracheal (B) inoculation with Ck/B/99 (filled squares) and Sw/B/98 (open squares) viruses are given from 1 up to 6 dpi. Mean virus titers of Ck/B/99 and Sw/B/98 are displayed as full and dashed lines respectively. ---: detection limit. Virus titres were significantly higher for Sw/B/98 than for Ck/B/99 (P<0.05) and after intranasal than after intratracheal inoculation with both viruses (P<0.05).

Figure 1A compares the nasal virus excretion curves of Ck/B/99 and Sw/B/98. Ck/B/99 was detected in nasal swabs of all pigs examined between 1 and 6 dpi, except for one of both pigs at 5 dpi. Virus titers were highly variable between pigs and ranged from 1.5 up to 5.5 log_10_ EID_50_/100 mg. The duration of virus excretion was comparable for Ck/B/99 and Sw/B/98 (P>0.05), but titers of the latter virus were significantly higher (P<0.05) and less variable.

To compare the organ tropism of both influenza viruses, samples from the upper and lower respiratory tract and selected extra-respiratory tissues were examined for virus replication. Ck/B/99 was isolated from the respiratory tract of all pigs except for one pig euthanized at 5 dpi. These pigs tested virus positive in several parts of the respiratory tract, but virus isolation rates and virus titers were highly variable between individual pigs as shown in Table 1. Ck/B/99 was isolated from 44 of the total 60 (73%) upper respiratory tract samples and from 22 of the total 42 (52%) lower respiratory tract samples. In the upper respiratory tract, virus was most frequently isolated from the olfactory part of the nasal mucosa and from the nasopharynx. In the lower respiratory tract, the virus did not replicate uniformly throughout all lung lobes. Unexpectedly, Ck/B/99 was also isolated from the brain stem of three out of 12 pigs, but not from the intestinal tract, spleen or serum. The ileum, colon, spleen and brain stem were examined with RT-PCR, but viral RNA was only detected in the three brain stem samples that were positive in virus titration (Tables 2 and 3). Sw/B/98 had a similar organ tropism as Ck/B/99 and infectious virus was recovered from the respiratory tract and brain stem only (Tables 2 to [Table pone-0006662-t003] 4). The virus was isolated from 22 out of 30 (73%) and 22 out of 24 (92%) samples of the upper and lower respiratory tract respectively. Virus isolation rates in the lower respiratory tract and virus titers in the upper and lower respiratory tract were significantly higher than for the Ck/B/99 virus (P<0.05). Using RT-PCR, viral RNA was detected in the brain stem, ileum and colon of some pigs (Table 2).

**Table 1:** Distribution of an H5N2 AIV in the respiratory tract of pigs after intranasal inoculation.

Tissue	Virus titers (Log_10_ EID_50_/gram) at … days post inoculation^a^											
	1		2		3		4		5		6	
	#1	#2	#3	#4	#5	#6	#7	#8	#9	#10	#11	#12
Nasal mucosa R^b^	3.3	3.7	2.8	4.3	<^e^	3.5	<	1.8	<	<	<	2.3
Nasal mucosa O^c^	2.2	2.3	2.5	3.5	3.3	5.5	2.5	3.6	<	3.3	2.5	5.6
Nasopharynx	4.6	4.3	3.5	3.5	<	3.8	1.8	3.8	<	2.8	2.3	3.5
Tonsil	4.8	2.3	3.1	<	1.6	2.3	<	3.6	<	2.2	<	<
Trachea	2.5	2.5	2.3	3.9	<	4.5	3.3	4.5	<	2.2	<	3.3
Lung A^d^	2.7	na^f^	5.4	na	1.2	na	3.0	4.5	<	3.8	<	2.7
Lung B^d^	4.5	na	3.0	na	<	na	<	2.5	<	<	<	2.7
Lung C^d^	<	<	<	4.0	<	8.3	<	3.5	<	4.5	<	4.5
Lung D^d^	6.0	3.5	<	3.9	<	6.0	1.9	<	<	3.0	<	<

a Virus titers are shown for each individual pig (#).

b Respiratory part of the nasal mucosa.

c Olfactory part of the nasal mucosa.

d Lung A apical+cardiac lung lobes right, Lung B diaphragmatic lung lobe right, Lung C apical+cardiac lung lobes left, Lung D diaphragmatic lung lobe left.

e <Below detection limit (1.5 log_10_ EID_50_/gram for the upper respiratory tract samples, 1.2 log_10_ EID_50_/gram for the lung samples).

f Na not available.

**Table 2:** Detection of avian and swine influenza viruses in extra-respiratory tissues.

Virus	Inoculation route	Total number of pigs	Number of positive pigs							
			Brain stem		Ileum		Colon		Spleen	
			VI^a^	PCR	VI	PCR	VI	PCR	VI	PCR
Ck/B/99	intranasal	12	3	3	0	0	0	0	0	0
Sw/B/98		6	3	5	0	1	0	4	0	0
Ck/B/99	intratracheal	12	0	0	0	1	0	0	0	0
Sw/B/98		12	0	3	0	3	0	3	0	2

a VI virus isolation.

**Table 3 pone-0006662-t003:** Kinetics of influenza virus detection in the brain stem after intranasal inoculation.

Virus	N pigs per timepoint	Number of positive pigs at … days post inoculation^a^											
		1		2		3		4		5		6	
		VI^b^	PCR	VI	PCR	VI	PCR	VI	PCR	VI	PCR	VI	PCR
Ck/B/99	2	1 (3.0)	1 (38.3)	1 (1.2)	1 (36.7)	1 (1.2)	1 (39.6)	0	0	0	0	0	0
Sw/B/98	1	0	1 (37.6)	1 (2.5)	1 (35.5)	1 (4.4)	1 (30.4)	1 (2.0)	1 (34.7)	0	1 (39.0)	0	0

a Individual virus titers (log_10_ ID_50_/gram) and ct values of the positive pigs are given between brackets.

b VI virus isolation.

**Table 4:** Distribution of an H1N1 swine influenza virus in the respiratory tract of pigs after intranasal inoculation.

Tissue	Virus titers (Log_10_ TCID_50_/gram) at … days post inoculation^a^					
	1	2	3	4	5	6
	#1	#2	#3	#4	#5	#6
Nasal mucosa R^b^	5.3	5.3	4.7	5.3	3.7	<^e^
Nasal mucosa O^c^	4.0	5.0	5.8	5.3	<	<
Nasopharynx	4.3	5.5	3.3	5.3	<	<
Tonsil	4.0	4.0	4.0	2.8	<	<
Trachea	5.7	6.3	6.7	6.5	5.3	<
Lung A^d^	2.5	8.2	7.0	7.7	6.0	4.7
Lung B^d^	2.6	6.3	6.5	6.5	5.0	<
Lung C^d^	2.5	5.5	6.7	5.7	4.5	<
Lung D^d^	2.3	5.5	6.3	6.5	5.3	4.3

a,b,c,d See table 1.

e <Below detection limit (1.9 log_10_ TCID_50_/gram for the upper respiratory tract samples; 1.7 log_10_ TCID_50_/gram for the lung samples)

**Figure 2:**
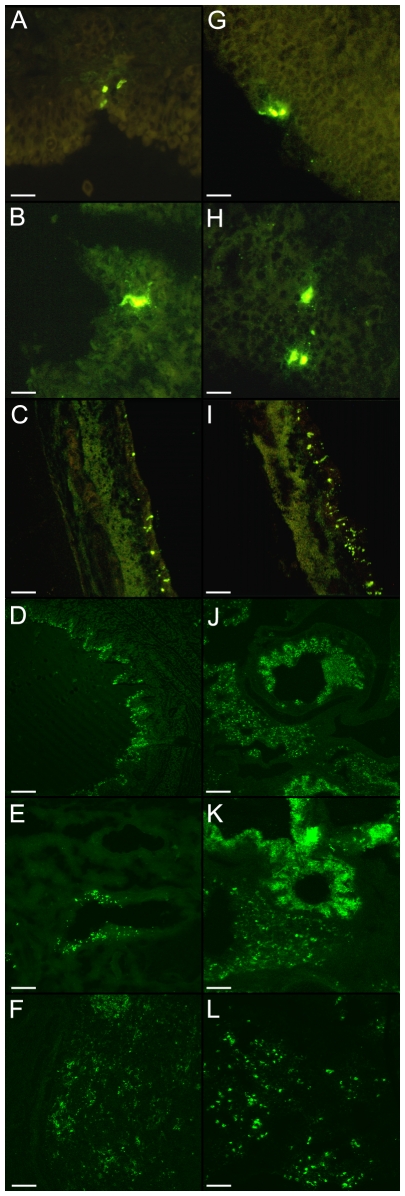
Viral antigen distribution of Ck/B/99 (left column) and Sw/B/98 (right column) throughout the porcine respiratory tract. The figure shows antigen positive cells in the respiratory (A,G) and olfactory part (B,H) of the nasal mucosa after intranasal inoculation, and in the trachea (C,I), bronchi (D,J), bronchioles (E,K) and alveoli (F,L) after intratracheal inoculation. Bars represent 12.5 µm (A,B,G,H), 25 µm (C,I,F,L) or 50 µm (D,E,J,K). Both viruses showed a similar distribution but the total number of positive sections was up to 100 times lower for the avian than for the swine virus. Positive sections also contained fewer positive cells with the avian virus, and we only show the sections with most antigen positive cells.

Immunofluorescence stainings were performed on all tissues of the respiratory tract and on the brain stem to confirm the results of virus titrations and to better define the tissue tropism and quantitative differences in replication between the avian and swine viruses. Ck/B/99 virus antigen positive cells were found in all tissues of the respiratory tract, but the numbers of positive samples and cells were minimal, especially in the upper respiratory tract (Figure 2). The nasal mucosa, nasopharynx and trachea occasionally exhibited single positive epithelial cells, covering less than 1% of the epithelium. The olfactory part of the nasal mucosa was more frequently positive than the respiratory part. In the tonsils, positive cells were mainly found in the diffuse lymphatic tissue and the germinal centers and occasionally as debris in the tonsillar crypts (Table 5). The lung samples tested more frequently positive than the upper respiratory tract tissues, and groups of positive cells were observed in the bronchioli and alveoli of a few pigs (Table 6). After inoculation with Sw/B/98, more positive samples were found at all levels of the respiratory tract. As with the Ck/B/99 virus, the nasal mucosa, nasopharynx and tonsils showed only single positive cells. In contrast with Ck/B/99, the trachea, bronchi, alveoli, and particularly bronchioli contained massive numbers of positive cells. Frequently up to 100% of the bronchiolar epithelium was positive. No viral antigen positive cells were found in the brain stem with either virus.

**Table 5:** Semi-quantitative assessment of swine and avian influenza antigen positive cells in the upper respiratory tract.

Virus	Inoculation route	Tissue	Extent of immunofluorescence at … days post inoculation^a^											
			1		2		3		4		5		6	
			% pos^b^	IF score^c^	% pos	IF score	% pos	IF score	% pos	IF score	% pos	IF score	% pos	IF score
Ck/B/99	intranasal	Nasal mucosa R^d^	0	-	0	-	13	±	13	±	13	±	0	-
		Nasal mucosa O^e^	0	-	100	±	75	+	38	±	50	±	50	±
		Nasopharynx	0	-	50	±	50	±	75	±	50	±	50	±
		Trachea	38	±	0	-	13	±	50	+	38	±	25	±
Sw/B/98	intranasal	Nasal mucosa R	0	-	50	±	100	±	100	±	75	±	0	-
		Nasal mucosa O	75	±	50	±	100	+	100	±	0	-	0	-
		Nasopharynx	50	±	100	+	25	±	100	±	50	±	0	-
		Trachea	0	-	75	±	100	++	100	++	100	+	0	-

a Results from one pig (Sw/B/98) or means of two pigs (Ck/B/99).

b Percentage of sections containing viral antigen positive cells.

c Immunofluorescence score; -: negative; ±: <1% epithelial cells positive; +: 1 to 10% positive; ++: >10 to 50% positive; +++: >50% positive.

d Respiratory part of the nasal mucosa.

e Olfactory part of the nasal mucosa.

**Table 6:** Semi-quantitative assessment of swine and avian influenza antigen positive cells in the lungs.

Virus	Inoculation route	Lung structures	Extent of immunofluorescence at … days post inoculation^a^											
			1		2		3		4		5		6	
			% pos^b^	IF score^c^	% pos	IF score	% pos	IF score	% pos	IF score	% pos	IF score	% pos	IF score
Ck/B/99	intranasal	Bronchi	0	-	0	-	0	-	0	-	0	-	0	-
		Bronchioli	0.4	+	0.1	+	7	+++	1	+++	0.3	+	0	-
		Alveoli		±		±		++		++		++		±
Sw/B/98	intranasal	Bronchi	0	-	15	++	22	++	69	+++	24	++	3	+
		Bronchioli	0	-	21	++++	6	+++	20	++++	8	+++	3	++
		Alveoli		-		+++		++		++		±		±
Ck/B/99	intratracheal	Bronchi	14	++	3	++	6	++	4	++	0	-	0	-
		Bronchioli	4	++	2	++	3	++	0.03	++	1	++	0.3	++
		Alveoli		++		++		++		++	1	++		+
Sw/B/98	intratracheal	Bronchi	100	++++	79	+++	83	+++	34	+++	5	++	0	-
		Bronchioli	77	++++	62	++++	48	++++	29	+++	12	++	0	-
		Alveoli		++		++++		+++		+++		++		-

a Results are means of two pigs; except for the Sw/B/98 intranasal group where only one pig was available.

b Percentage of bronchi and bronchioli containing viral antigen positive cells.

c Immunofluorescence score; -: negative; ±: <0.5% of epithelium positive (single cells); +: 0.5 to 1% positive; ++: >1 to 10% positive; +++: >10 to 50% positive; ++++: >50% positive.

### Intratracheal inoculation of pigs with an avian H5N2 or swine H1N1 influenza virus

Similar experiments were performed using an intratracheal inoculation method. This method avoids the physical barriers in the upper respiratory tract and reproducibly induces the typical acute symptoms of swine influenza with swine influenza viruses.

Unlike the intranasal inoculation, intratracheal inoculation with Ck/B/99 or Sw/B/98 induced depression and increased abdominal thumping in all pigs for 1 up to 3 dpi and these symptoms were clearly most pronounced with Sw/B/98. Gross lesions were restricted to the lungs, but were more prominent than after intranasal inoculation. Typical influenza lesions were observed in all but one pig with both viruses, but they were less severe with Ck/B/99 (1 to 33% lung involvement) than with Sw/B/98 (8 to 58% lung involvement). The right lung half tended to be most affected.

Figure 1B compares the nasal virus excretion curves of Ck/B/99 and Sw/B/98. Ck/B/99 was detected in nasal swabs of only one pig, at 3 dpi. Sw/B/98 was detected in nasal swabs of all pigs between 3 and 5 dpi. For both viruses, nasal virus shedding was lower and shorter after intratracheal than after intranasal inoculation (P<0.05).

The organ tropism of Ck/B/99 was more profoundly affected by the route of inoculation than that of the swine virus. Ck/B/99 was isolated from the respiratory tract of all pigs except for one pig euthanized 5 dpi. The virus distribution within the respiratory tract differed from that observed after intranasal inoculation as shown in Table 7. Fifteen out of 60 (25%) samples of the upper respiratory tract and 29 out of 42 (69%) samples of the lower respiratory tract were virus positive. In the upper respiratory tract, the nasal mucosa remained completely virus negative in all pigs and the virus was most frequently recovered from the trachea. In the lungs, Ck/B/99 was more evenly distributed among all lung lobes and virus titers were generally higher than those obtained after intranasal inoculation (P>0.05). Extra-respiratory samples were negative by virus isolation, but a single ileum sample was positive by RT-PCR (Table 2). Sw/B/98 was more frequently isolated from the upper respiratory tract than Ck/B/99 with 40% of the 60 samples being virus positive (P<0.05) (Table 8). Virus isolation rates and virus titers in the lower respiratory tract were comparable between the two viruses (P>0.05), with 75% of the 48 samples being positive for Sw/B/99. No infectious Sw/B/98 virus was detected in the extra-respiratory samples, but several samples tested positive in the RT-PCR (Table 2).

**Table 7:** Distribution of an H5N2 AIV in the respiratory tract of pigs after intratracheal inoculation.

Tissue	Virus titers (Log_10_ EID_50_/gram) at … days post inoculation^a^											
	1		2		3		4		5		6	
	#1	#2	#3	#4	#5	#6	#7	#8	#9	#10	#11	#12
Nasal mucosa R^b^	<^e^	<	<	<	<	<	<	<	<	<	<	<
Nasal mucosa O^c^	<	<	<	<	<	<	<	<	<	<	<	<
Nasopharynx	<	<	<	<	6.2	<	2.3	2.3	<	<	<	<
Tonsil	<	<	<	<	3.7	<	2.3	<	2.2	<	<	1.5
Trachea	6.5	2.2	5.0	2.2	5.7	<	4.3	4.8	2.1	<	<	<
Lung A^d^	6.7	5.3	7.0	na^f^	6.9	na	5.7	5.0	4.9	<	6.3	<
Lung B^d^	6.8	7.2	5.2	na	6.5	na	5.9	5.0	3.0	<	<	<
Lung C^d^	6.8	na	5.3	<	6.3	4.2	3.0	4.7	4.5	<	1.2	<
Lung D^d^	5.9	na	5.3	<	6.3	3.7	3.9	5.5	<	<	<	<

a,b,c,d,e,f See table 1.

**Table 8:** Distribution of an H1N1 swine influenza virus in the respiratory tract of pigs after intratracheal inoculation.

Tissue	Virus titers (Log_10_ TCID_50_/gram) at … days post inoculation^a^											
	1		2		3		4		5		6	
	#1	#2	#3	#4	#5	#6	#7	#8	#9	#10	#11	#12
Nasal mucosa R^b^	<^e^	<	<	<	<	2.0	<	3.8	3.3	<	<	<
Nasal mucosa O^c^	<	<	<	<	<	<	<	<	2.0	<	<	<
Nasopharynx	2.8	2.3	5.0	5.8	<	5.0	5.0	6.0	3.7	<	<	<
Tonsil	2.0	<	4.6	2.7	<	<	<	<	<	2.3	<	<
Trachea	8.7	8.0	7.3	7.0	6.0	6.8	6.3	6.8	<	<	<	<
Lung A^d^	3.5	7.5	7.0	8.3	7.5	na^f^	6.5	6.3	3.8	<	<	<
Lung B^d^	6.5	7.0	5.8	7.5	5.7	6.7	4.7	5.5	1.7	<	<	<
Lung C^d^	8.5	8.7	5.5	5.7	5.5	6.7	1.7	6.5	5.7	<	<	<
Lung D^d^	7.2	7.7	4.5	7.0	4.5	7.0	4.3	5.3	3.2	<	<	<

a,b,c,d,e, f See table 4.

Ck/B/99 viral antigen positive cells were undetectable in the upper respiratory tract of most pigs. The nasopharynx and tonsils rarely showed single positive cells, and a few pigs had more positive cells in the trachea (up to 10% of the epithelium) and fluorescing debris in the lumen (Figure 2). Numbers of positive sections and immunofluorescence scores were higher in the lungs than in the upper respiratory tract (Table 6). After inoculation with Sw/B/98, the nasal mucosa, nasopharynx and tonsils also showed few and solitary positive cells. Sections of the trachea and lungs in contrast, were usually positive, up to 80% of the epithelial lining stained positive and there was abundant fluorescing cellular debris in the lumen. Immunofluorescence scores in the lung were also considerably higher for Sw/B/98 than for Ck/B/99.

## Discussion

Pigs are susceptible to AIVs and potential sources for the emergence of pandemic viruses of avian origin but the pathogenesis of AIVs in the pig has hardly been studied. We have therefore undertaken a classical comparative study of the pathogenesis of a LP H5N2 AIV and an H1N1 swine influenza virus. We used two different inoculation routes and performed sequential virologic examinations on different tissues to compare the extent and site of virus replication throughout the course of infection. Though the swine influenza virus is of avian origin, it is representative of typical swine-adapted influenza viruses. It has characteristic amino acid substitutions in the receptor binding site of its HA that are also present in “classical” swine H1N1 viruses circulating in the US [27]. Furthermore, its internal genes are identical to those of European H3N2 and H1N2 swine influenza viruses, which have a similar pathogenesis as the avian-like H1N1 virus [25].

The swine and avian viruses replicated in the entire respiratory tract and both viruses showed the strongest tropism for the lungs. The major difference was the lower efficiency of replication of the AIV as shown by lower virus titers and immunofluorescence scores at all levels of the respiratory tract. The AIV clearly caused a productive infection of a similar duration as the swine virus but it appeared to be hampered in its capacity to spread within the respiratory tract. This was illustrated by the more profound effect of the inoculation route on the distribution of the AIV compared to the swine influenza virus. After intranasal inoculation the AIV did not replicate uniformly throughout all lung lobes and it was even not consistently isolated from the upper respiratory tract. The intratracheal inoculation resulted in higher lung virus titers and a more even distribution of virus throughout the lung in the present experiment. Still, even the intratracheal inoculation did not invariably result in homogenous virus titers in the lungs in other experiments with Ck/B/99 or other LP H5 AIVs [28], [29]. Poor virus release from the few cells that do become infected with the AIV could be a contributing factor to this inefficient virus spread. Furthermore, the extreme individual variation in AIV replication between pigs that lack any specific anti-influenza immunity suggests an important role of host factors. The response of cytokines and cells of the innate immune system, and their effects during AIV infection, are ill-defined and may vary strongly between individuals. Respiratory mucus has been shown to interfere with influenza infection via decoy receptors and its composition also varies between individuals [30].

While we did not compare their exact cell-tropism, we found both the avian and the swine influenza virus in epithelial cells along the entire respiratory tract. The AIV, however, infected proportionally fewer cells than the swine virus at all levels of the respiratory tract. Consequently, AIV infected cells were most scarce in the respiratory part of the nasal mucosa, which is likely a major site of deposition of virus particles under natural circumstances. The lack of susceptible cells at this site may in part explain the relatively lower susceptibility of pigs to avian than to swine influenza viruses, as well as the lower amounts of virus in nasal secretions of AIV infected pigs. Indeed, the results of the intratracheal inoculations indicate that most of the virus in nasal swabs results from local virus production in the nasal mucosa. Parallel studies in corresponding organ cultures of the porcine respiratory tract in our laboratory have also identified the nasal mucosa and trachea as being least susceptible to AIV infection: 24 hours after inoculation of the organ cultures AIV yields were highest in the bronchial and alveolar cultures and significantly lower in the nasal and tracheal cultures. Interestingly, this corresponds with an almost exclusive detection of the presumed AIV receptor, Sia α2,3 Gal, in the smaller bronchioli and alveoli (submitted for publication). In studies with fixed tissue sections of the porcine respiratory tract HP H5N1 and LP H5N9 and H6N1 viruses attached preferentially to alveoli while there was minimal binding to the trachea and bronchi [26]. At the same time our *in vivo* study demonstrates that the restricted replication of the AIV cannot be explained purely on the basis of the Sia receptor distribution. Despite the uniform presence of Sia α2,3 Gal in the pig lung, the AIV did not replicate uniformly in the lung. Also, some extent of infection of the upper respiratory tract clearly occurred in the absence of the AIV receptor. Most important, the avian and the swine influenza virus clearly differed more in their replication capacity than in their tropism. This may reflect the limited fitness of the internal genes of AIVs to support replication in porcine cells. Two recent studies with LP H5N2 and HP H5N1 viruses have convincingly demonstrated that the PB2 and/or other internal genes restrict the replication of these viruses in the pig [8], [31].

The brain stem was the single extra-respiratory tissue that was positive by virus isolation and RT-PCR after intranasal inoculation with avian or swine influenza virus in several pigs. These pigs had no neurological symptoms and we could not detect viral antigen positive cells in their brains. Still, preliminary *in vitro* tests confirmed the ability of the Sw/B/98 virus to infect neurons of the porcine trigeminal ganglion via their axons (unpublished). Similar observations have also been made with a HP H5N3 AIV and murine sensory neurons from the dorsal root ganglia [32]. The influenza viruses may thus have reached the brain stem of the pigs by invading the afferent fibers of the cranial nerves after replication in the nasal mucosa. Again, such a ‘neuronal pathway of virus spread has been demonstrated in the mouse model for an HP H5N1 virus which appears to use extensions of the vagal and trigeminal nerves to spread from the respiratory tract to the brain stem and later to the cerebral cortex [33]. The hypothesis of neuronal spread in pigs is consistent with the lack of detectable viremia and with the association between virus titers in the olfactory part of the nasal mucosa and those in the brain stem. Of course it does not explain the exclusive detection of viral RNA in the brain stem of some pigs after intratracheal inoculation with the swine influenza virus, because these pigs lacked virus replication in the nasal mucosa. It is possible that these pigs had an undetectable viremia which could account for the detection of viral RNA in their brain and spleen. In humans influenza virus positive cells in the brain have been occasionally demonstrated in fatal cases of infection with HP H5N1 or conventional H1N1 or H3N2 viruses [34]–[38]. Many of these cases had excessive virus replication in the lungs and, less frequently, virus spread to extra-respiratory organs, but mechanisms of virus spread to the brain remain unclear. Pigs may offer a valuable model to investigate these mechanisms, as well as the factors that may facilitate virus spread to the brain.

A few intestinal samples contained low amounts of viral RNA, but no infectious virus. The absence of infectious virus corresponds with the inability of Kida et al. [15] to isolate LPAIVs from rectal swabs of experimentally infected pigs. We assume that the viral RNA does not result from active virus replication in the intestinal tract, but from the ingestion of virus-loaded respiratory secretions. In this case, the virus will be diluted during the digestive processes and gradually lose its infectivity due to the many adverse conditions and low pH in the gastrointestinal tract. This can also explain why viral RNA was only detected in pigs with relatively high amounts of virus in the respiratory tract. It is intriguing that experimental respiratory inoculation of chickens with Ck/B/99 resulted in virus isolation from the caeca, but the reasons for this difference remain obscure.

Overall, our data do not reject the theory of the pig as an intermediate host for AIVs but highlight the strong species barrier to infection of pigs with a wholly avian H5N2 virus. Our study remains merely descriptive, but it will serve as a basis to further explore the cellular pathogenesis of AIVs in the pig, as well as their neuro-invasiveness. Based on the present findings, we have also started to use experimental LP H5 infection of pigs as a model to examine heterologous protection induced by candidate H5 vaccines or other immunisation strategies [28], [29]. For this purpose, the pigs are challenged both intranasally and intratracheally to ensure maximal virus replication as well as clinical symptoms. By using a LP H5 challenge virus we circumvent some of the limitations that are inherent to experimental infections with HP H5 viruses. Furthermore, there are no indications for a higher virulence or more invasive character of HP viruses in pigs. Experimental intranasal inoculations of pigs with four HP H5N1 viruses induced only minimal symptoms and pathology and a strict respiratory infection. Virus titers in the lungs were higher than those in the upper respiratory tract and significantly lower than those produced by swine influenza viruses [16], as in our study.

## Materials and Methods

### Ethics statement

The experiments were authorized and supervised by the Ethical and Animal Welfare Committee of the Faculty of Veterinary Medicine of Ghent University.

### Influenza viruses

The LPAIV isolate A/chicken/Belgium/150/99 H5N2 (Ck/B/99) was isolated from tissue samples from poultry in Belgium. The swine influenza virus A/swine/Belgium/1/98 H1N1 (Sw/B/98) was isolated from an outbreak of acute respiratory disease in Belgium. Both viruses were grown on 11-day-old embryonated chicken eggs and used at the third egg passage.

We previously reported the complete coding sequence of the haemagglutinin (HA), neuraminidase (NA), matrix (M) and nucleoprotein (NP) gene segments of Ck/B/99 [29], confirming that the virus is representative for LP H5 AIVs circulating in Europe in the late nineties. Sw/B/98 belongs to the avian-like H1N1 lineage.

### Animals and experimental design

Forty-two four-week-old conventional pigs were purchased from commercial herds free of antibodies to any influenza A virus as shown in a competitive anti-influenza A nucleocapsid ELISA (ID-VET). The pigs were allocated to three groups of 12 pigs and one group of six pigs. All groups were housed in separate biosafety level-2 HEPA filtered isolation units. One group of 12 pigs and one group of six pigs were inoculated intranasally with Ck/B/99 and Sw/B/98 respectively. The intranasal inoculations were performed with 7.0 log_10_ EID_50_ in 3 ml of phosphate-buffered saline (PBS) (1.5 ml per nostril). Two groups of 12 pigs each were inoculated intratracheally with Ck/B/99 and Sw/B/98 respectively at a dose of 7.5 log_10_ EID_50_ in 3 ml of PBS. The intratracheal inoculations were performed with a 20 Gauge needle through the skin cranial to the sternum. From 1 until 6 dpi, one or two pigs from each group were euthanized. At the time of necropsy, gross pathological examinations were performed, and the following samples were collected for virological examinations: nasal mucosa respiratory part (i.e. nasal turbinates), nasal mucosa olfactory part (i.e. ethmoid labyrinth), nasopharynx, tonsils, trachea, lung, brain stem, spleen, intestines (duodenum, jejunum, ileum, colon and rectum) and serum. Because preliminary experiments had shown an uneven distribution of the AIV in the pig lung, we collected 4 different lung samples: 1) right apical and cardiac, 2) right diaphragmatic, 3) left apical and cardiac, and 4) left diaphragmatic lung lobes. To prevent cross-contamination seperate sterile instruments were used to sample the respiratory and extra-respiratory tissues of each pig, and instruments were decontaminated between each sample. Nasal swabs were collected daily from 0 dpi until euthanasia, starting with six pigs per group.

### Clinical monitoring and assessment of gross lung lesions

All pigs were monitored daily for general (depression, anorexia) and respiratory (coughing, dyspnoea, abdominal thumping, tachypnoea) symptoms from 3 days before inoculation until euthanasia. Percentages of gross lung lesions was calculated as an average of the percentages of the dorsal and ventral lung surface area showing tissue consolidation.

### Virus titration

Nasal swabs and all tissue samples were examined by virus titration. Nasal swabs were put into 1 ml of transport medium (PBS supplemented with 10% fetal bovine serum, 100 IU/ml penicillin and 100 µg/ml streptomycin) and mixed vigorously for 1 hour at 4°C. The medium was clarified by centrifugation and used for titration. Tissue samples were weighed and ground in PBS containing 10 IU/ml penicillin and 10 µg/ml streptomycin to obtain 10 or 20% tissue homogenates. The homogenates were clarified by centrifugation and used for titration. All samples of Ck/B/99 inoculated pigs were titrated on 11-day-old embryonated chicken eggs. Briefly, eggs were inoculated with 200 µl of 10-fold sample dilutions by the allantoic route. After 72 h of incubation at 37°C, allantoic fluid was tested for hemagglutinating activity with 0,5% chicken erythrocytes. All samples of the Sw/B/98 inoculated pigs were titrated in Madin Darby Canine Kidney (MDCK) cells. Briefly, MDCK cells were seeded in 96-well cell culture plates at a concentration of 250000 cells per ml. After three days of incubation, the cells were inoculated with 10-fold dilutions of the samples. The cells were observed daily for the presence of cytopathic effect and after seven days virus titers were calculated by the method of Reed and Muench. Previous examinations failed to show significant differences in virus titers in eggs compared to MDCK cells in respiratory tract samples of Ck/B/99 inoculated pigs. Virus identification was performed by hemagglutination inhibition tests using monospecific post-infection swine sera against Ck/B/99 and Sw/B/98.

### RT-PCR

Samples of the ileum, colon, spleen and brain stem were examined by real-time RT-PCR using a newly developed PriProET RT-PCR targeting the matrix gene of influenza A virus. The assay is based on a PriProET probe (5′ CCCAGTGAGCGAGGACTGCAGCGT-Cy5 3′) and a set of published primers [39] with the modification that the reverse primer has a FAM fluorophor at the 5′ end. Total RNA was extracted from the samples using the Boom-silica method as previously described [40]. Five µl of extracted RNA was tested using the RNA Ultrasense RT-PCR kit (Invitrogen) in 25 µl reactions containing 100 nM forward primer and 500 nM of each FAM-primer and PriProET probe. One-step real time RT-PCR was performed in a Mx3005p (Stratagene) using the following cycle program conditions: 50°C for 15 min, 95°C for 2 min, 55 cycles of 95°C for 15 s, 55°C for 15 s and 75°C for 15 s. This was immediately followed by generation of a probe melting curve, starting from 40°C and ending at 95°C for confirmation of specific amplification. The FRET FAM/Cy5 fluorescence data obtained at the annealing step were used to assign a cycle threshold (ct) value to each sample using a fixed threshold of 100. All samples with a ct value of 40 or higher were considered negative. Each panel of samples was tested individually and standardised swine and AIV RNA samples were included in each RT-PCR set-up to monitor inter-assay variability.

### Immunofluorescence

All samples of the respiratory tract were examined in immunofluorescence stainings, extra-respiratory tissues were only examined when positive in virus isolation. The samples were embedded in methyl cellulose and stored at −70°C until use. Tissue sections of 7 µm thickness were fixed in acetone and incubated with a monoclonal antibody targeting the influenza A virus nucleoprotein (HB65, ATCC, 1/50) and fluorescein isothiocyanate labelled goat-anti-mouse monoclonal antibody (F-2761, Invitrogen, 1/200). The lung samples were subsequently incubated with anti-desmin monoclonal antibodies (Clone D33, Dako, 1/50) to visualize the smooth muscle tissue surrounding the bronchi and bronchioli, followed by Texas Red-conjugated goat anti-mouse monoclonal antibody (T-6390, Invitrogen, 1/100). The extent of virus replication in the upper and lower respiratory tract was assessed semi-quantitatively. For the upper respiratory tract, 16 sections of each sample were evaluated. For each sample, the number of positive sections was counted and the number of fluorescing cells in the epithelium was scored (Table 7). For the lungs, 48 sections of each pig, spread over all lung lobes, were examined. The total number of bronchi (defined as airways surrounded by cartilage) and bronchioli (not surrounded by cartilage) were counted for each pig, as well as the number showing fluorescence. The IF score is an estimate of the number of fluorescing cells in the epithelium of bronchi, bronchioli and alveoli (Table 8).

### Statistical analysis

Virus titers in nasal swabs and the duration of excretion were compared for the avian and the swine virus by Wilcoxon rank-sum tests. Standard two-sample t-tests were used to compare virus titers and virus isolation rates in the respiratory tract. Differences were considered significant when P<0.05.
